# Hey, *You*! The Importance of Pragmatics in Localizations of *Mass Effect* in French and Spanish

**DOI:** 10.1177/15554120231218046

**Published:** 2023-12-12

**Authors:** Alexander Stainton, Seán G. Roberts, Stephanie Rennick

**Affiliations:** 1 School of English, Communication, and Philosophy, 2112 Cardiff University, Cardiff, UK; 2 Department of Communications, Media and Culture, 7622 University of Stirling, Stirling, UK

**Keywords:** localization, translation, mass effect, pragmatics, politeness

## Abstract

The localization of video game dialog for diverse audiences is challenging because of differences in linguistic features between languages and pragmatic norms between cultures. For example, localizers must decide how to translate the English second-person singular pronoun “you” into languages that have a pragmatic distinction between formal and informal pronouns (e.g., “vous” and “tu” in French). These distinctions are used in social interaction to signal politeness, respect, and social distance, which are important elements that shape player experience in role-playing games. We analyze the dialog from French and Spanish localizations of *Mass Effect* and show they have strikingly different strategies for translating pronouns. French mostly uses formal pronouns while Spanish mostly uses informal pronouns. We explain how these differences affect player experience and argue that effective localization requires a clear strategy for dealing with pragmatics. We conclude by making practical suggestions for how game creators can better support localization.

## Introduction

Localizing media involves a culturally sensitive conversion from a text designed for one audience to suit another. This involves several challenges, including dealing with linguistic and cultural differences (Bernal-Merino, 2020). For role-playing games, the heart of this challenge is a consideration of pragmatics: how people use language to navigate social relationships (requesting, demanding, apologizing, insulting, complimenting), in contrast to the literal forms they produce. A central principle in pragmatics is that people are expected to adhere to norms of behavior which linguists call “politeness” (Brown & Levinson, 1987): speakers should avoid publicly threatening the social standing of their interlocutor by avoiding interactions which suggest that their interlocutor is not liked or not autonomous. According to Brown and Levinson, face-to-face communication in all cultures broadly follows this principle. However, the norms of polite behavior differ between cultures and the tools for expressing politeness differ between languages. For example, while the second-person pronoun “you” is a fairly neutral word in English, a French speaker is forced to choose whether to signal informality and familiarity by using “tu,” or signal respect and distance by using “vous.”

These pragmatic differences mean that the localization of dialog is difficult because it needs to preserve both the semantic meaning and the implied social interaction in a way that makes sense for the intended audience. While this is true for many media, it is particularly challenging for role-playing video games for several reasons. Firstly, modern role-playing games can contain much more dialog than a typical film or television program—often in the hundreds of thousands of words—making the translation task resource intensive. Furthermore, due to player choices and branching dialogs, they include a great range of possibilities for what characters might intend, ranging across extremes from polite compliments to death threats, all of which need to be communicated to the player. Secondly, while film and television translators typically have full access to the final product, game localizers are frequently unable to actually play the final game (e.g., because the game is only available in development versions during development, or there are tight deadlines between finishing the game code and shipping in multiple locales), meaning that crucial context is not always available (see Rivas Ginel & Theroine, 2022a). Finally, pragmatic meanings are critical for the role-playing genre. For example, video game dialog options mostly vary at the pragmatic level and are critical to player experience (Anon, submitted). Unsystematic or ad-hoc localization strategies can lead to costly mistakes: video game localization specialist Pierre Techoueyres describes a case where there were differences in how individual members of a localization team translated English “you” to French “tu” and “vous.” The inconsistency was jarring to players, and the localizers needed to retranslate 10,000 words (cited in Rivas Ginel & Theroine, 2022a, p. 360). In general, failure to meet these challenges may result in stilted and awkward dialog, which in turn may affect player immersion and ultimately player satisfaction.

This project considers the first and third *Mass Effect* games, and investigates how these games have been converted into other languages in terms of formality and politeness, looking specifically at formal and informal personal pronouns. The *Mass Effect* games are role-playing action games with a futuristic setting. The player controls a military commander named Shepard (who the player can choose to be male or female) who is tasked with saving the galaxy from various alien threats. Interactive dialog is a key part of the role-playing experience, with player options having significant impacts on relationships with nonplayer characters (NPCs) and the plot.

The *Mass Effect* games have been localized into several languages, including French and Spanish, both of which have “full” localizations of all audio dialog and game text (Ray, 2019). Both of these languages have different second-person pronouns depending on the formality of the address. This article focuses on how neutral English *you* has been localized. Do French and Spanish demonstrate different patterns of formality in their usage of second-person pronouns, all from the same English *you*? Or does one language prove to prefer a more formal localization? What might be the causes of these differences? We address these questions, and find that the two localizations have strikingly different strategies: the Spanish localization rarely uses formal pronouns and the French localization rarely uses informal pronouns. This creates different player experiences in domains such as communicating social hierarchy and romantic relationships. We argue that the differences are not simple reflections of wider cultural differences, but specific localization strategies. We conclude that an understanding of pragmatics is critical for effective localization in video game dialog and make practical recommendations for how game creators can support this.

## Literature Review

All languages have various features for negotiating social relations and norms for how linguistic distinctions signal politeness (Brown & Levinson, 1987). This concept is not necessarily about being “nice,” but instead concerns cultural conventions for how interlocutors should interact without threatening each other's social status. The aim of this paper is to investigate *Mass Effect* 1 and 3 (Bioware, 2007, 2012) and their localization into French and Spanish, with a specific focus on pragmatic disparities in the usage of formal versus informal second-person pronouns. Around a third of languages have politeness distinctions in personal pronouns (Helmbrecht, 2013). For example, in French, the informal second-person singular pronoun is “tu.” “Vous,” on the other hand, doubles as the plural second-person pronoun (which is neutral in terms of formality) and the formal counterpart of *tu*, able to be used in singular contexts (Harris, 1988, p. 221). *Tu* is typically reserved for family-members, friends, or those of inferior status or position (e.g., a master addressing their servant, Harris, 1988, p. 221). Castilian Spanish, by contrast, has five second-person pronouns. *Tú* and *usted* are the informal and formal singular pronouns respectively. *Ustedes* is the formal second-person plural pronoun, while *vosotros* and *vosotras* are the informal second-person plural pronouns (Stewart, 2003). *Vosotras* is reserved for groups of females (Green, 1988, p. 96).

Politeness norms are, of course, culturally relative. However, various studies suggest that French would be more likely to use a formal pronoun than Spanish for the same context. For example, compared to French speakers, Spanish speakers typically use more direct politeness (e.g., including both speaker and hearer) and positive politeness (e.g., signaling affiliation, making jokes, attending to interests, Bataller, 2013; Marsily, 2015). Furthermore, Spanish-speaking cultures often score more highly on cross-cultural measures of informality and looseness than French-speaking cultures (e.g., Gelfand et al., 2011).

Achieving culturally sensitive translations of the pragmatics of politeness is a central challenge for translation (Hatim & Mason, 2014, pp. 76–97; House, 2006, 2018; Nord, 1997). This is also true for video game localization, though there are few studies of issues with pragmatics in this domain. Exceptions include Touiserkani (2015) which studies the localization of the *Half-Life* games into Persian and mentions that politeness strategies differ in 18% of translated lines (e.g., moving from less direct requests to more direct demands). Rivas Ginel and Theroine (2022b) assess various automated translation tools for localization and identify specific problems for translating English to French, including mistranslation of formal and informal pronouns.

There may be practical considerations for localization strategies. For example, Rivas Ginel and Theroine (2022b) suggest that “the use of ‘vous’ in its polite form allows the translator to avoid specifying the gender,” facilitating translation when the gender of the interlocutors might vary according to player choices. However, it is not clear to us how this would save work: there are no cases where using “vous” instead of “tu” would change the requirements for gendered language (Grevisse & Lenoble-Pinson, 2009, p. 322). Furthermore, there are several cases of lines differing only in English gendered pronouns for the player character, showing that game creators are willing to invest in supporting this kind of variation.

There are increasing calls for a consideration of pragmatics in localization (e.g., Casado Valenzuela, 2018) but localization is not always applied with a clear understanding of the linguistic theory of pragmatics. For example, Jiménez-Crespo (2011) emphasizes the importance of considering pragmatics in the quality assessment of software localization. However, they categorize errors in politeness under “stylistics” rather than “pragmatics.”

In summary, while there is evidence that pragmatics is a source of difficulty for localization, there are few systematic studies which investigate the extent of differences in localization strategies involving pragmatics and politeness. This study aims to address this gap in the literature.

## Method

The dialog data for *Mass Effect 1* and *Mass Effect 3* were taken from the Video Game Dialogue Corpus (Rennick & Roberts, 2024). This includes dialog in English, French and Spanish which are aligned at the level of lines of dialog. Localizations were not available for *Mass Effect* 2. The dialog was processed in R (R Core Team, 2022) and R studio (RStudio Team, 2021), using the *quanteda* library for managing corpus data (Benoit et al., 2018). The game data for *Mass Effect* includes duplicate lines of dialog in different files. These were recognized by ID number and removed to leave only unique lines of dialog. Lines of dialog were removed if they had no linguistic content in any one of the three languages considered. Some of the main characters are named Liara T'Soni and Aria T’Loak. These surnames were removed to avoid confusion with the French contraction of “te” (“t’”).

There were several considerations when looking at formality. Firstly, only personal pronouns were explicitly searched for in the corpus, as they are the most straightforward indicator of the formality of address. However, it would not be a fair comparison in French to compare the words “vous” and “tu,” given the fact that while “tu” is only a subject pronoun in French, “vous” is simultaneously a subject, (in)direct object, tonic and reflexive pronoun, which means that “*vous”* would almost certainly outnumber “tu” even in the situation where formality and informality were on par. Therefore, the informal equivalents of “*te*,” “*toi,”* and “*t’”* were also searched for and taken with “*tu”* to calculate the informal French usage.

The total list of French tokens searched for therefore was: *tu, te, toi, t’,* and *vous*. The total list of Spanish tokens searched for was: *tú, te, ti, vosotros, vosotras, os, usted,* and *ustedes*. The Spanish object and reflexive counterparts of “*usted(es)”* were not searched for as they are hard to distinguish from definite articles (e.g., “*la”*) and third-person pronouns (e.g., “*se,*” “*lo*,” etc., Green, 1988, p. 95). Additionally, Spanish is a pro-drop language while French is not (i.e., pronouns may be omitted in Spanish where they can be inferred by the grammar or pragmatics). We discuss these complications in the results section.

The log-likelihood test and χ^2^ test were the statistical measures used to indicate the significance of the differences in frequency. The log-likelihood test is a standard test in corpus linguistics which compares whether the difference between two-word frequency counts (e.g., two different words in the same corpus or the same word in two different corpora) is significantly greater than one would expect by chance, given that we know frequency distributions are on an exponential scale (Rayson & Garside, 2000). The χ^2^ test compares the observed frequencies in a contingency table to what would be expected by chance. We use it to compare whether there is a significant difference in the distribution of formal and informal pronouns between French and Spanish.

To help understand the results from the video game dialog, we compared them to the frequency of pronouns in film dialog, taken from the parallel Open Subtitles corpus in Spanish and French (Lison & Tiedemann, 2016). These were chosen because they represent the largest corpora with a similar design in both languages that also contain spoken language in a dramatic, scripted genre. Similar corpora are not available for other video game dialog.

## Results

The final *Mass Effect* dataset contained 46,835 lines of English dialog translated into French and Spanish. This included 481,906 words of English, 490,783 words of French and 483,133 words of Spanish. The word “you” (and the colloquial “ya”) appeared in English 15,255 times in 13,185 lines. Tables 1 and 2 show the frequency of different forms of second-person pronouns in Spanish and French respectively.

**Table 1.** Frequency of Different Informal and Formal Second-Person Pronouns in Spanish.

Spanish	Case	Informal	Freq	Formal	Freq
Singular	Nominative	Tú	1,108	usted	28
	Prepositional	ti	571		
	Object	Te	3,314	lo/la/le,se	*
Plural	Nominative/prepositional	vosotr[o/a]s	87	ustedes	1
	Object	Os	196	los/las/les, se	*
Total			**5,276**		**29**

*These words were not counted since they are difficult to tell apart from nonsecond-person constituents.

**Table 2.** Frequency of Different Informal and Formal Secong-Person Pronouns in French.

French	Case	Informal	Freq	Formal	Freq
Singular	Nominative	tu	490	vous	13,473^†^
	Object	te, t'	270		
	Disjunctive	toi	70		
Total			**830**		13,473

†This frequency includes both singular formal and plural neutral uses (see discussion).

There are three main results. First, Spanish uses far more informal pronouns than formal pronouns. Second, French uses far more formal pronouns than informal pronouns. Finally, and as a logical consequence, French uses a significantly greater proportion of formal pronouns than Spanish, χ^2^(1)  =  15,832, *p* < .001.

The frequencies above have some caveats, which we now address. Firstly, not all cases of “vous” are strictly formal; it can be used as a neutral plural pronoun as well. Automatic part-of-speech taggers were applied to the data, but none could reliably differentiate singular from plural “vous.” To estimate the proportion of neutral plural uses, a random sample of 100 French lines containing “vous” was manually categorized for a person by a fluent French speaker. Using the context of the whole line as well as the English and French translations, lines were coded as singular, plural, or ambiguous. Ninety-two percent of these were singular (92 singular, six plural, two ambiguous due to the context). Additionally, since the French dialog has a parallel translation in Spanish, we can use the frequency of singular and plural second-person verbs in Spanish as an estimate of the proportion of address to one or more individuals. Frequencies for common verbs that clearly differed in singular and plural forms were obtained (eres/sois, estás/estáis, tienes/tenéis, eras/erais, dices/decís, quieres/queréis, haces/hacéis, miras/miráis, vas/vais, sigues/seguís). Ninety-four percent of these Spanish second-person verbs are singular in the data (2,800 singular forms vs 184 plural forms). So the majority of cases of “vous” are likely to be singular formal pronouns. This is perhaps not surprising given that most dialogs in *Mass Effect* take place between two individuals (the player character and an NPC). In any case, even if 8% of “vous” were plural neutral, this would not affect any of the three main results above.

The second caveat is that the formal Spanish Object case was not counted (lo, la, le, los, las, les, se). For example, “las” appears 3331 times. An automatic part-of-speech tagger suggested that 6% of these (200) were pronouns. This number may still include a mix of second- and third-person pronouns, but even if they were all second-person formal pronouns, and this number was similar for each of the seven object cases, this would not be enough to overturn the three main results above. Furthermore, the main results hold if we just look at the subject and tonic pronouns (“*tú,” “ti,” “vosotros,” “vosotras,”* appear 4,510 times, and *“usted,” “ustedes”* appear 29 times). This is significant given the fact that even though Spanish is a pro-drop language, “*usted”* and “*ustedes”* are the only second-person pronouns that are sometimes required to disambiguate a sentence since these pronouns employ third-person verbal conjugations and pronouns, whereas “*tú,*” *“vosotros,”* and “*vosotras”* verbal forms are always unambiguous. Even so, these formal pronouns appear significantly less than the informal pronouns.

In summary, there are large differences in localization strategies between French and Spanish. In order to understand whether this is due to broad cultural differences or is specific to this video game localization, the next section runs a similar analysis of film dialog.

### Comparison to Film Dialog

Tables 3 and 4 show the frequency of Spanish and French pronouns in the Open Subtitles corpus of film dialog. For Spanish, all informal pronouns were used significantly more in *Mass Effect* than in Open Subtitles (except for “te”) while the formal pronouns were used significantly less in *Mass Effect* than in Open Subtitles. For French, the opposite pattern holds: informal pronouns were used significantly less frequently in *Mass Effect* compared to Open Subtitles and “vous” was used significantly more frequently in *Mass Effect* compared to Open Subtitles. The log-likelihood statistics show that the differences between *Mass Effect* and Open Subtitles are larger for French than for Spanish. This difference is not observed for other pronouns. For example, the first-person pronoun “je” is only used 10% more in *Mass Effect* than in Open Subtitles, while “vous” is used 214% more and “tu” appears 1,213% less.

**Table 3.** The Frequency of Various Pronoun Words in Spanish in Mass Effect and a General Film Subtitle Corpus.

Word	Frequency in mass effect	Frequency per million words in mass effect	Frequency in open subtitles	Frequency per million words in open subtitles	Log-likelihood	*p*-value
tú	1,108	2,293.4	1,662,657	1,663.3	+ 102.95	<.0001
ti	571	1,181.9	861,378	861.7	+ 51.41	<.0001
te	3,314	6,859.4	8,327,391	8,330.7	−133.60	<.0001
vosotr [o/a]s	87	180.1	68,734	68.8	+ 59.92	<.0001
os	196	405.7	181,342	181.4	+ 98.71	<.0001
usted	28	58.0	1,341,639	1,342.2	−1,064.63	<.0001
ustedes	1	2.1	323,566	323.7	−300.59	<.0001

The columns show the word, the raw frequency in mass effect, the relative frequency (per million words) in mass effect, the raw frequency in open subtitles, the relative frequency (per million words) in open subtitles, the log-likelihood test statistic, and the associated *p*-value.

**Table 4.** The Frequency of Various Pronoun Words in French in Mass Effect and a General Film Subtitle Corpus.

Word	Frequency in mass effect	Frequency per million words in mass effect	Frequency in open subtitles	Frequency per million words in open subtitles	Log-likelihood	*p*-value
tu	490	998.4	7,033,501	12,108.7	−8,455.61	<.0001
te, t'	270	550.1	3,557,298	6,124.1	−4,167.84	<.0001
toi	70	142.6	1,338,724	2,304.7	−1,731.84	<.0001
vous	13,473	27,452.1	7,459,863	12,842.7	+ 6,123.05	<.0001

The columns show the word, the raw frequency in mass effect, the relative frequency (per million words) in mass effect, the raw frequency in open subtitles, the relative frequency (per million words) in open subtitles, the log-likelihood test statistic, and the associated *p*-value.

The French and Spanish Open Subtitles corpora have a small number of parallel lines of dialog translated from the same original text. These were searched to find formal or informal second-person pronouns in French that had parallel translations into formal or informal pronouns in Spanish (see Table 5). The results show significant variation in alignment, χ^2^(1)  =  11.2, *p* < .001. French pronouns are nearly twice as likely to be aligned with formal Spanish pronouns than informal French pronouns. However, overall, French pronouns of either kind are most likely to be aligned with Spanish informal pronouns. This suggests that Spanish is more informal than French in film dialog, as we observe in the video game dialog. Still, the difference is much greater for the dialog in *Mass Effect*. For example, the ratio of informal to formal in French film dialog is only 1.6:1, while in *Mass Effect* it is 1:16.2. In summary, the patterns in *Mass Effect* do not seem to simply mirror more general cultural differences. To explore this further, we look at specific differences between the French and Spanish localizations.

### Social Hierarchy

This section digs deeper into the pragmatic uses of formal and informal pronouns in *Mass Effect*. Some patterns relate to social hierarchies. For example, in Spanish, formal “usted” is used by some soldiers to their superiors, though this is not consistent. “Usted” is also used in public PA system announcements, reflecting politeness norms in the real world (Peterson, 2023, p. 29). Some individual characters seem to use the variation to signal changes in social relations. For example, a reporter in the first game initially uses “usted” while interviewing Shepard. However, once Shepard upsets the reporter by either being evasive or outright berating the reporter, then the reporter switches to “tú” and turns hostile. In the third game, she only uses informal pronouns while addressing Shepard, perhaps remembering their previous encounter. Butt et al. (2018, p. 137) note that contempt is a typical motivation for using “tú” where “usted” might be expected.

An interesting pattern is found in the relations between biological and technological characters. In French, while “*tu”* and its declensions were less common than “*vous*,” it was routinely used by humans and other sapient biological alien characters while addressing technology such as AI, virtual intelligence (VI), robots and other interactive software. Meanwhile, these same technological entities would address people using “*vous*.” Similarly, in Spanish, almost every use of “*usted”* occurs in the context of technology. Much like French, the Spanish VI and AI of *Mass Effect* use formal pronouns to address people, but people use informal pronouns to address AI in return.

This imbalance reflects an inherent hierarchy between people and machines, with machines clearly not considered to be deserving of the same respect from people, that people are considered to deserve from their very own creations. This is in keeping with some of the narrative themes explored in the game (Faller, 2016).

The notable exception to this rule is the VI Avina, which is a learning VI that eventually gets to “know” the playable protagonist. In the first game, Avina addresses Shepard using formal pronouns in Spanish, for example during a guided tour: “Está usted cerca de la base de la Torre de la Ciudadela” (“You are standing near the base of the Citadel Tower”). But by the end of the game, Avina is addressing Shepard using informal pronouns: “Respetamos tu privacidad, comandante Shepard” (“We respect your privacy, Commander Shepard”). In contrast, Shepard uses an informal register when talking to Avina throughout. This shift in formality coming from Avina is evidence of a shift in the relationship and dynamic between Avina and Shepard, to the point where Avina and Shepard seem to view each other more as equals after a considerable time of knowing each other and interacting.

A similar change happens in French. However, this time it is Shepard who changes their language. For example, in *Mass Effect* 1, Shepard asks Avina a question using formal pronouns “Vous êtes une personne ou un programme?” (“So are you a person or a program?”). By the third game, Shepard is using informal pronouns: “Tu as des informations sur le Purgatoire?” (“Do you have any information on Purgatory?”). Indeed, the frequency of Shepard using “tu” when talking to Avina increases steadily over the course of the two games (first quarter of lines: 0%, second quarter: 27%, third quarter: 33%, fourth quarter: 40%). In contrast, Avina never uses informal pronouns. Once the formality shifts between Shepard and Avina it remains consistent until the end of the series. This consistency might indicate a conscious effort on the part of the localizers. However, our main point is that the relationship dynamic between Shepard and Avina is different in the two localizations. Although the relationship becomes more informal in both localizations, in Spanish it is Avina who changes their behavior while in French it is Shepard who changes their behavior. Given that Shepard begins in a dominant social position and Avina begins in a subordinate position, the two localizations provide quite different messages about how social equality is achieved.

### Romance

**Figure 1.**
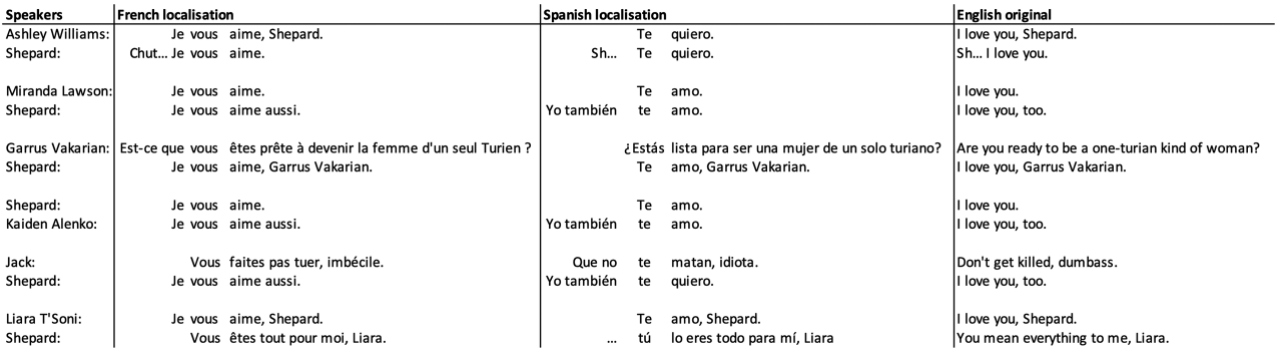
Formal vous between romantic partners in French, Spanish and the original English.

A slightly less expected pattern is found in romantic interactions in French. The theory of politeness suggests that informal “*tu”* would be used between close relations, such as lovers. However, Shepard and his/her lovers use formal “*vous”* pronouns in conversation, as shown in Figure 1. This usage is consistent across several different romantic partners. This does not align with the expected behavior from pragmatic theory and seems to differ from other contexts. For example, in the Open Subtitles corpus of film dialog, “je vous aime” is attested, but is nearly 10 times less frequent than the informal “je t’aime” (9,754 times vs 83,342 times, log-likelihood  =  −66,600, *p* < .001). Furthermore, Spanish localization always uses informal pronouns (and verbs in the case of “*estás”*) in the same situations.

### Differences Between Characters

In a more exploratory analysis, we looked at the variation in pronoun formality between characters in the French localization (the Spanish localization has very few formal pronouns to study). For each of the 30 characters with more than 2,000 words of dialog, the frequency of informal and formal pronouns in their own speech was calculated using the same methods as above. Table 6 shows the five most formal speakers and the five most informal speakers.

**Table 5.** The Frequency of Occurrences of Second-Person Pronoun Types in Parallel Translations From the Open Subtitles Corpus.

	Spanish informal	Spanish formal
French informal	701	92
French formal	437	98

**Table 6.** Formal and Informal Pronoun Frequency in the Total Speech of Different Characters in French.

Character	Formal frequency	Informal frequency	Informal proportion	Total words	Log-likelihood
Admiral Hackett	233	0	0.0%	10,397	−323.01
Samantha Traynor	134	0	0.0%	4,596	−185.76
Legion	101	0	0.0%	4,463	−140.02
Donnel Udina	98	0	0.0%	3,370	−135.86
Dr Chakwas	77	0	0.0%	2,558	−106.74
…					
Engineer Adams	48	3	5.9%	2,158	−47.88
Steve Cortez	109	7	6.0%	4,027	−107.93
James Vega	235	19	7.5%	10,494	−217.05
Jeff “Joker” Moreau	200	18	8.3%	7,283	−177.95
Miranda Lawson	63	6	8.7%	2,017	−54.88

The “informal proportion” shows the informal frequency as a percentage of formal  +  informal frequencies. “Total words” shows the total number of words of dialog in the data for a given character. The “Log likelihood” shows the statistic comparing formal frequency to informal frequency, with numbers further from zero indicating a more extreme imbalance. The rows are sorted by informal proportion then by log-likelihood.

The most formal speakers all have clear character motivations for using signaling formality, respect, or social distance. Hackett and Udina have high-ranking official roles and Legion is an antagonistic AI whose formality signals social distance. Chakwas is the ship's medic and she appears to be older than Shepard. While Traynor becomes a potential romantic partner for female Shepard, she begins as a new member of the ship's crew and is deferential to her superior.

Similarly, most informal speakers have clear motivations to be informal. Lawson, Vega, and Cortez are some of Shepard's core squad members and also potential romantic partners. Additionally, Lawson begins as Shepard's superior. Joker (Shepard's loyal quippy pilot) and Vega (an experienced marine) both have little regard for the strictures of authority. Vega in particular has a very informal attitude, using nicknames for Shepard and flirting with a female Shepard despite her being his superior. Interestingly, Engineer Adams has a close relationship with the ship's AI, tying into the first theme above.

Therefore, formality distinctions seem to be exploited by the localizers in a way that emphasizes differences between characters. An alternative hypothesis is that there would be a difference between genders, since previous research has found that female dialog in video games is more likely to be polite than male dialog (Rennick et al., 2023). However, there was no significant difference between male and female usage of informal pronouns in the full data (permutation test *p*  =  .61). So, at least impressionistically, the pronoun measure captures a more general difference in character formality, opening the possibility of study characters and their development through their dialog. Interestingly, since the Spanish dialog has so few formal pronouns, it misses out on being able to communicate differences in character by this particular linguistic variable.

## Discussion

This study compared the use of formal and informal pronouns in two localizations of video games in the *Mass Effect* series. The source language, English, does not include these distinctions while they are required for Spanish and French. This requires the localization team to interpret the pragmatic context and make decisions about how to encode this extra aspect of politeness. Interestingly, the two localizations studied here differ strikingly in their solutions to localizing the same source material. Second-person pronouns in French are mostly formal while in Spanish they are mostly informal. While there are technical challenges to estimating the true frequency of second-person pronouns (homonyms with different parts of speech, pronoun dropping), we argue above that these are unlikely to change the central result given the magnitude of the differences between localization strategies.

The study found several examples of choices about formal and informal pronouns that affect the depiction of the relationships between the player character and NPCs. These included norms for humans interacting with AI and changes in pronoun use following a change in relationship dynamics. These are good examples of localization strategies that take advantage of a language's specific features to enhance the player's experience. They exploit the difference between formal and informal pronouns to achieve several important effects. Firstly, they can communicate interpersonal relationship dynamics to the player, such as becoming more familiar (Shepard and the VI Avina) or more hostile (Shepard and the reporter). Secondly, some of the choices about pronouns help develop themes important to the games’ narrative, such as the nature of the relationship between humans and AI. Finally, it can enhance the immersive interaction between players and NPCs. In several cases, appropriate selection of pronouns aligns with politeness norms (i.e., the linguistic content aligns with the pragmatic intention). This helps give the impression that the NPCs have a pragmatic mind that is sensitive to what they are *doing with language*, rather than just puppets that are saying words. Rennick and Roberts (2021) argue that this is a key part of creating immersive game dialogs.

In contrast, there were some uses of formal pronouns in inappropriate settings. For example in French, formal “vous” is consistently used between romantic partners. This differs from the Spanish localization and from norms in French film dialog. A mismatch between linguistic politeness and pragmatic closeness is likely to be interpreted as unrealistic behavior by players. In contrast, there was very little use of formal options in Spanish. For example, although characters vary in their pronoun formality in French in tune with their general character, the Spanish dialog had very little variation between characters. This seems like a missed opportunity to use linguistic variation for characterization.

It is possible that the difference between localizations is driven by wider differences in formality between Francophone and Hispanophone cultures. However, the size of the differences in the Mass Effect are considerable, and the same differences were not found in an analysis of film dialog, which suggests that culture is not the sole explanation. Alternatively, the patterns might be explained by automatic translation, since tools such as Google Translate have known problems with translating English pronouns to other languages (e.g., Tourmen & Hoffmann, 2022). For example, Rivas Ginel and Theroine (2022b) found errors in the automatic translation of pronouns from English to French in the game *The Devil's Womb*. However, the first *Mass Effect* game was released in 2007 with the French and Spanish localizations released in 2008. This is only 2 years after the launch of Google Translate, which would be unlikely to have been an effective translation method at that time, especially given the science fiction terminology in *Mass Effect*.

Therefore, it seems like the differences found in this study are more likely to be due to idiosyncratic localization strategies for *Mass Effect* in particular. We do not know whether these were deliberate decisions by the localization team, or whether the localization team were even aware of the patterns. Still, the size of the differences suggests that there was some difference in strategy between the two localization teams and that this might lead to quite different player experiences of the pragmatic relations between characters.

## Conclusion

We have shown that consideration of cross-cultural variation is important for effective localization of video game dialog, particularly for the role-playing genre where interpersonal relations are central to the player experience. If the role of localizers is to provide an immersive and authentic experience, it follows that they may need to add interpretations that are not present in the source but which the target language and culture demand. Doing this without radically departing from the originally intended experience is difficult and, as we have shown, can be realized in quite different ways. Here we suggest three tentative strategies for making sure players get the intended experience in multiple locales.

The first strategy is to systematically mark up lines of dialog with information on pragmatic intention. This kind of information is already present in some games. For example, the dialog script for *Dragon Age: Origins* (Bioware, 2009) includes directions to the voice actors, and several involve pragmatics (see below, with directions in square brackets):

- “Alistair”: “Mmm …  no, I don't think I can take that.” (politely refusing a gift)
- “Alistair”: “Right. Well, how do you … woo them? Is there a … technique? Or …” (he says “woo” because he's too polite to say “have sex,” very awkward at asking this)
- “Crimson Oars”: “Oh, you’re threatening us? We are veterans of many battles. Who are you, hm?” (Greatly amused, proud at himself—his insult shouldn’t sting too much—more posturing)
- “Kardol”: “Wondering what your life would be like if you had stayed in Orzammar? Few others of our kind would have you.” (Not insulting, just realistic. “You ask about us because we're the only ones who would have let you stay.”)

This kind of information is also valuable to localizers. However, the comment system for *Dragon Age: Origins* does not cover every line and does not use systematic categories. A more systematic markup for pragmatic intention could be used, similar to how each line in *The Elder Scrolls IV: Oblivion* (Bethesda Game Studios, 2006) is tagged with an emotion for use with the facial animation system. Various formal categorization systems exist for pragmatics in conversation, such as the Switchboard system (Calhoun et al., 2010).

**Figure 2.**
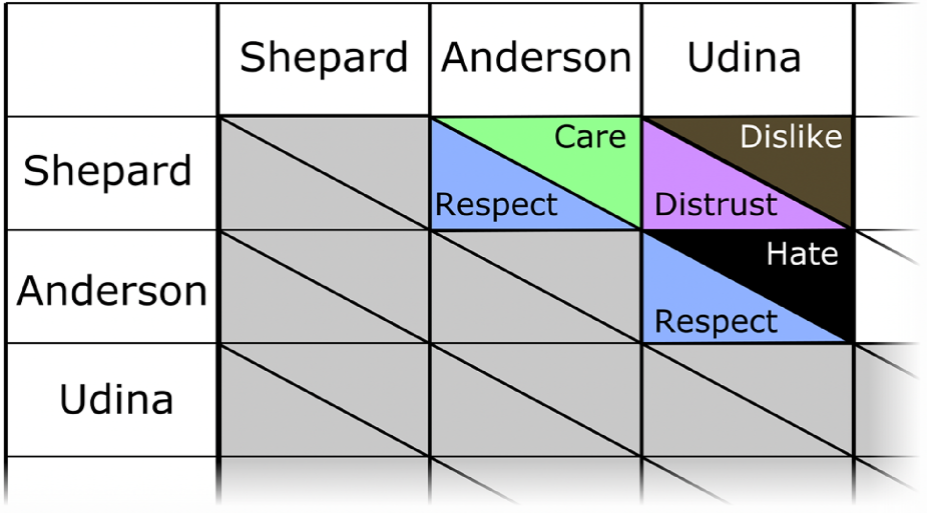
A hypothetical relationship chart for tracking pragmatic relations between characters.

The second strategy is to create development resources that make explicit the social relationships between characters in terms of social distance, hierarchy, and respect. This could help make sure that politeness relations are communicated through language consistently within and between localizations. Figure 2 shows a possible chart format for recording bidirectional social relations between characters in *Mass Effect*. Fans have created alternative ego-centered relationship charts (e.g., [bargu], 2020).

The third strategy is to have explicit linguistic localization strategies surrounding politeness. This could include an explicit strategy for translating formal and informal pronouns. This may be particularly important in games with fictional settings. For example, agreeing on what the politeness norms are and how they are expressed in an alien species, or the intention behind archaic language in a Medieval fantasy setting (e.g., use of “thou” and “ye” to reflect Middle English politeness distinctions, Crystal, 2004). This may be particularly difficult for games which include fictional languages. For example, in *Mass Effect*, Tali uses the word “bosh'tet” as a swearword in the fictional language of Khelish (an example of the “Pardon My Klingon” trope), but fans disagree about what the equivalent English insult would be, ranging from “jackass” to “bitch” to “motherfucker” (Ace_Of_No_Trades, 2021). This kind of information could be formalized in a wiki or design “Bible” to help deliver the intended player experience. For example, by giving paradigm responses for each category in Brown and Levinson's typology of response types.

Another possible solution for supporting localization that many game creators are increasingly interested in involves automatic machine translation and large language models like chatGPT (Rivas Ginel, 2022). However, given that tools based on large language models operate on overt forms rather than pragmatic intentions, we think it is unlikely that they will produce consistent and appropriate localization of pragmatics in dialog (e.g., Barattieri di San Pietro et al., 2023). At the very least, extra contextual information like in the three solutions above would be required for large language models to operate effectively, and there are additional ethical and legal issues with using AI-generated text (e.g., Ray, 2023).

Future research could extend the analyses here to other localizations of *Mass Effect* (German, Italian, Russian, Polish, Japanese), or other localized games. Critical to these studies is the availability of standardized and comprehensive texts from video games. The Video Game Dialogue Corpus is an expanding resource that can meet this need (Rennick & Roberts, 2024).
